# Irradiation induces cancer lung metastasis through activation of the cGAS–STING–CCL5 pathway in mesenchymal stromal cells

**DOI:** 10.1038/s41419-020-2546-5

**Published:** 2020-05-07

**Authors:** Zhiyuan Zheng, Shanfen Jia, Changshun Shao, Yufang Shi

**Affiliations:** The First Affiliated Hospital of Soochow University, State Key Laboratory of Radiation Medicine and Protection, Institutes for Translational Medicine, Soochow University Medical College, Suzhou, China

**Keywords:** Cancer microenvironment, Mesenchymal stem cells

## Abstract

Emerging evidence indicates that mesenchymal stromal cells (MSCs) have an important role in cancer metastasis. Although tumor microenvironment, which includes MSCs and immune cells, can be altered by ionizing radiation (IR), whether irradiation can promote metastasis through MSCs remains unclear. Using the lung colonization model of transplanted 4T1 breast cancer cells, we found an increased lung metastasis in mice exposed to ionizing radiation, even when the thorax was shielded during whole-body irradiation. This radiation-induced lung metastasis can be replicated using irradiated MSCs. cGAS–STING signaling pathway was found to be activated in MSCs, accompanied by upregulation of type I interferon-related genes, including chemokine CCL5. Disruption of cGAS–STING signaling in MSCs abolished their pro-metastatic effect. Deletion of CCL5 in MSCs also abrogated the pro-metastatic effect endowed by IR. Furthermore, we showed that the lung pro-metastatic effect of irradiated MSCs required the presence of macrophages. Our results indicate that radiation-induced alterations in distant mesenchymal stromal cells facilitate cancer metastasis.

## Introduction

Cancer metastasis, consisting of dissemination and secondary colonization of cancer cells, is the major cause of cancer-related death. Radiation therapy is widely used for the management of cancer^1^. Almost half of the cancer patients receive radiotherapy^1^. However, radiation therapy was shown to promote tumor metastasis in some mouse models^2^. Moreover, there is increasing evidence showing that radioresistance is not only attributed to tumor cells themselves, but also to the complex biological interactions between the tumor and its microenvironment. Meanwhile, radiation can results in remodeling in normal tissues, which may facilitate the initiation, invasion and metastasis of cancer cells^3^. However, how irradiation-induced alterations in tissue microenvironment may affect the colonization of cancer cells in distant organs remains poorly understood.

Mesenchymal stem cells (MSCs) exist in many tissues and have a critical role in maintaining tissue homeostasis. MSCs also serve as important components of tumor microenvironment due to their readiness to be recruited by tumors from both nearby and distant locations^4^. However, it is still unclear whether irradiated cells, especially MSCs in tissue microenvironment, can affect colonization of cancer cells in untargeted organs.

cGAS is an important cytosolic nucleic acid sensor and can be activated by double-stranded DNA (dsDNA)^5^. cGAS activation generates the cyclic dinucleotide cyclic GMP–AMP (cGAMP), which in turn induces a type I interferon response via STING^6–8^. cGAS–STING signaling was recently demonstrated to be critically involved in tumor development^6^. However, there have been conflicting reports whether the activation of cGAS–STING signaling inhibits or promotes tumor progression^9,10^. Moreover, the previous studies of cGAS–STING signaling in cancer are largely focused on tumor cells. Because the ubiquitous MSCs are relatively mobile and incur DNA double-strand breaks upon exposure to ionizing radiation (IR), we speculated the cGAS–STING signaling may become activated in MSCs as well in response to IR and contribute to the colonization of cancer cells in distant (untargeted) organs. We tested this using a mouse model of lung colonization of inoculated breast cancer cells.

We found that irradiation-induced metastasis is through MSCs and irradiated MSCs can facilitate metastasis to the lung. The cGAS–STING axis activated in irradiated MSCs is required for the pro-metastatic effect of the irradiated MSCs.

## Results

### Radiation promotes breast cancer metastasis

**Fig. 1:**
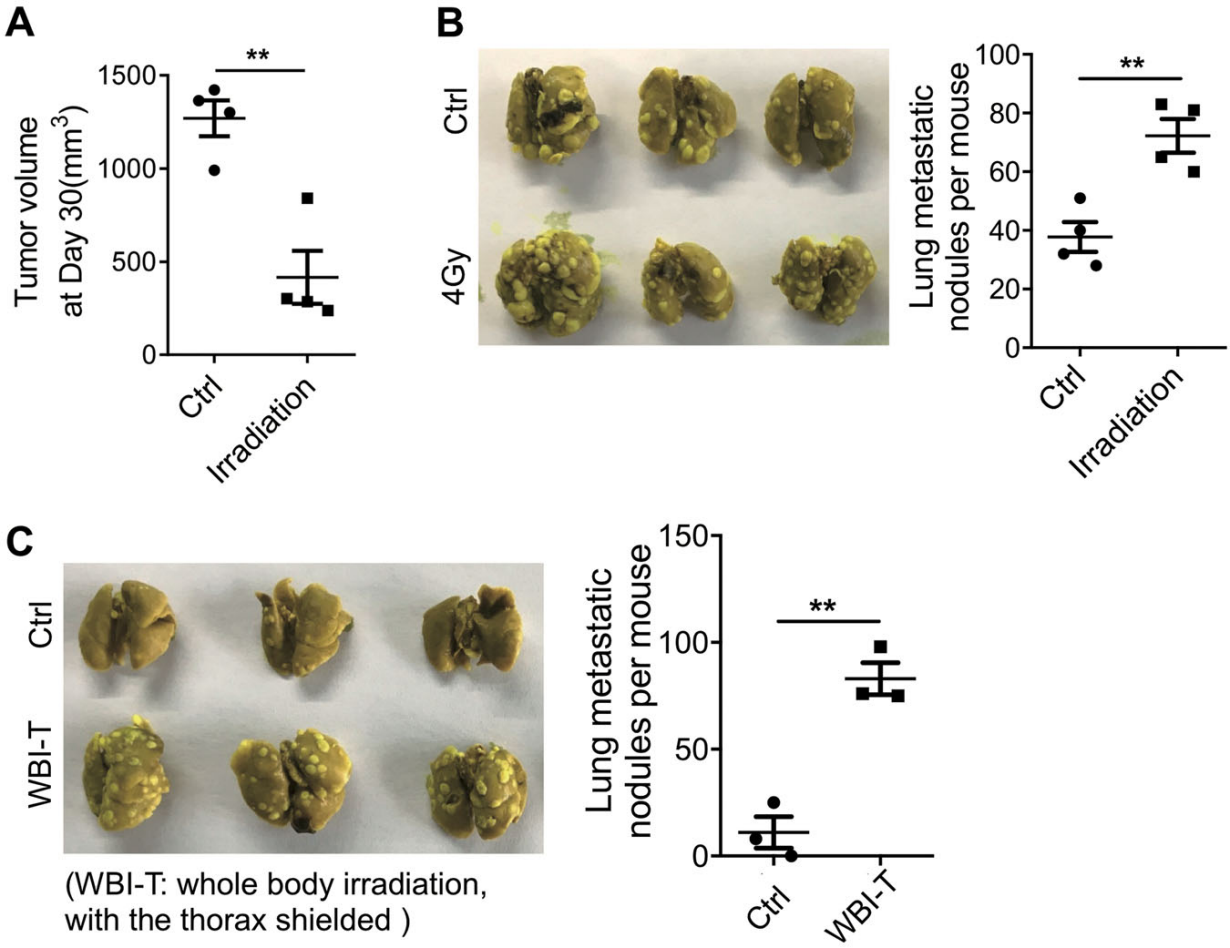
Local irradiation promotes lung metastasis of 4T1 cells. **a**, **b** BALB/c mice were subcutaneously injected with 4T1 cells (4 × 10^5^), 10 days later the tumor sites were irradiated (4 Gy) with X-ray. The tumor volume (**a**) and metastatic nodules (**b**) were recorded after 30 days. *n* = 4 for each group. **c** BALB/c mice were whole-body irradiated (4 Gy), but with the thorax shielded (WBI-T), and 4T1 cells (5 × 10^4^) were injected via tail vein within 24 h. Metastatic nodules were counted after 14 days. *n* = 3 for each group. All experiments in this figure were repeated at least three times. **p < 0.01.

Although studies performed in animal models indicate that cancer-targeted irradiation may promote tumor metastasis^11^, how irradiation may promote metastasis still remains unclear. Here, we studied the effect of radiation on lung metastasis of inoculated 4T1 mouse breast cancer cells. We inoculated 4T1 cells subcutaneously in BALB/c mice and 10 days later subjected the tumor area to irradiation (4 Gy). The tumor mass formed by 4T1 cells could be significantly reduced by local radiation (Fig. 1a). However, the radiation resulted in more metastatic nodules in the lung (Fig. 1b). This result indicated that while irradiation reduced primary tumor mass, it resulted in more lung metastasis. Because more metastasis occurs in unexposed lungs after tumor-targeted irradiation, one possibility we speculated is that irradiation may have altered the pulmonary microenvironment remotely so that the lungs become more accommodative to the circulating tumor cells. We tested this by exposing the mice to whole-body irradiation, but with the thorax shielded (WBI-T), and then injecting 4T1 cells via tail vein. Interestingly, this irradiation scheme also resulted in a remarkable increase in the number of metastatic nodules in the lung (Fig. 1c), supporting that the pro-metastatic effect of irradiation is systemic, not local in the lung.

### Irradiated MSCs promote metastasis

**Fig. 2:**
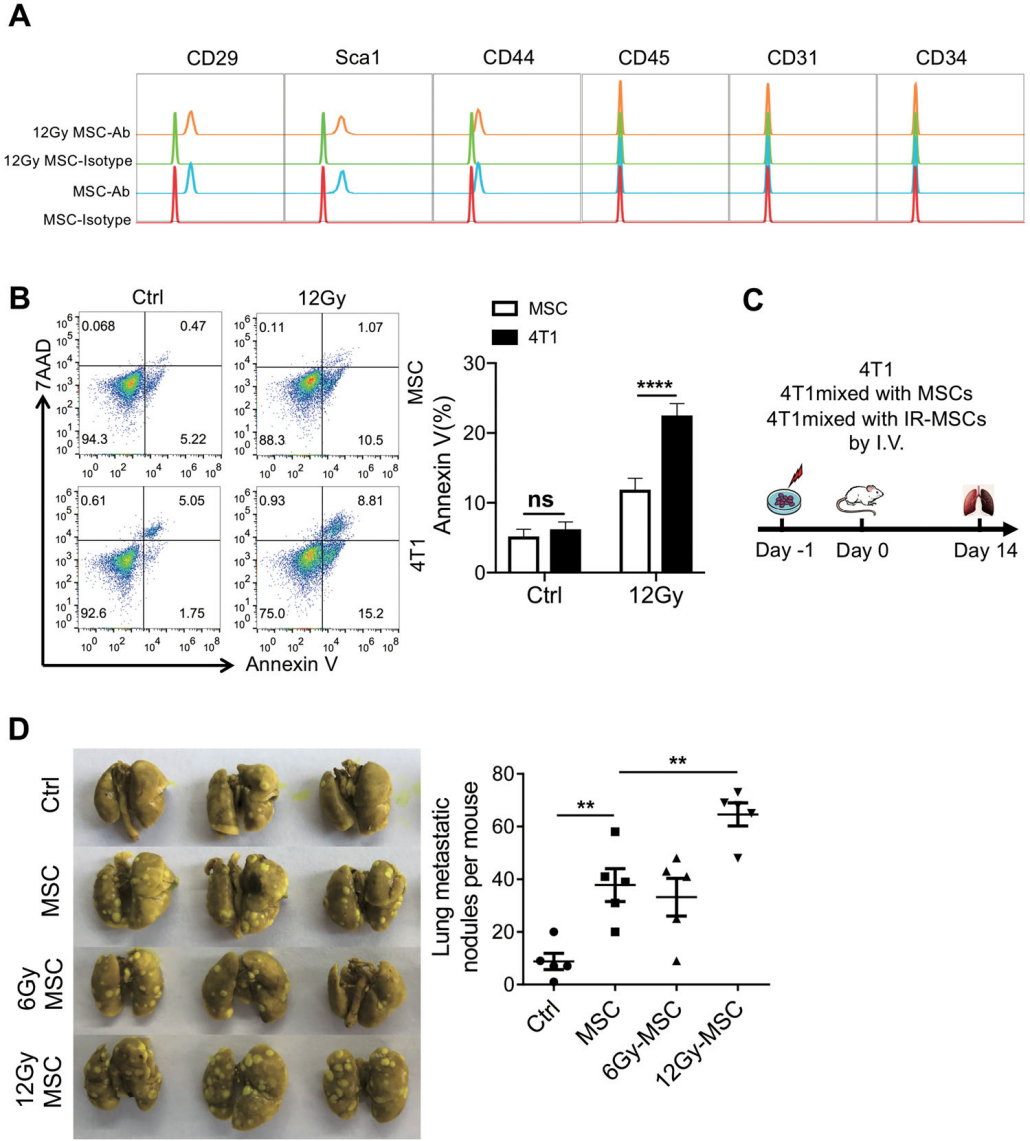
Irradiated MSCs promote lung metastasis of 4T1 cells. **a** MSCs and irradiated MSCs (12 Gy) were analyzed by the surface markers using flow cytometry. **b** 4T1 cells and MSCs were irradiated (12 Gy) and cultured for 24 h before the apoptosis ratios were analyzed by flow cytometry. **c**, **d** Schematic diagram of experimental protocol (**c**). MSCs were cultured for 24 h after irradiation (6 Gy or 12 Gy), and then were co-injected with 4T1 cells (5 ×10^4^) into BALB/c mice via tail vein. Metastasis lung tumor nodules were counted after 14 days (**d**). *n* ≥ 4 for each group. All experiments in this figure were repeated at least three times. **p < 0.01, ***p < 0.001; ns not significant.

Because hematopoietic cells are sensitive to IR and usually undergo massive apoptosis, whereas MSCs are resistant to apoptosis, we next tested whether MSCs could mediate the pro-metastatic effect of IR. We isolated MSCs from bone marrow and characterized their surface markers, observing that irradiated MSCs expressed the same surface markers in comparison to control MSCs (Fig. 2a). When exposed to the same dose of irradiation (12 Gy), MSCs were much more resistant to irradiation than 4T1 cells (Fig. 2b). When co-injected with 4T1 cells into BALB/c mice via tail vein, both the irradiated and control MSCs could increase the number of metastatic nodules in the lung. However, the MSCs irradiated with 12 Gy exhibited a much more pronounced pro-metastatic effect (Fig. 2c, d). These results indicated that the pro-metastatic effect of MSCs could be enhanced by IR.

### cGAS–STING signaling participates in irradiated MSCs-mediated metastasis

**Fig. 3:**
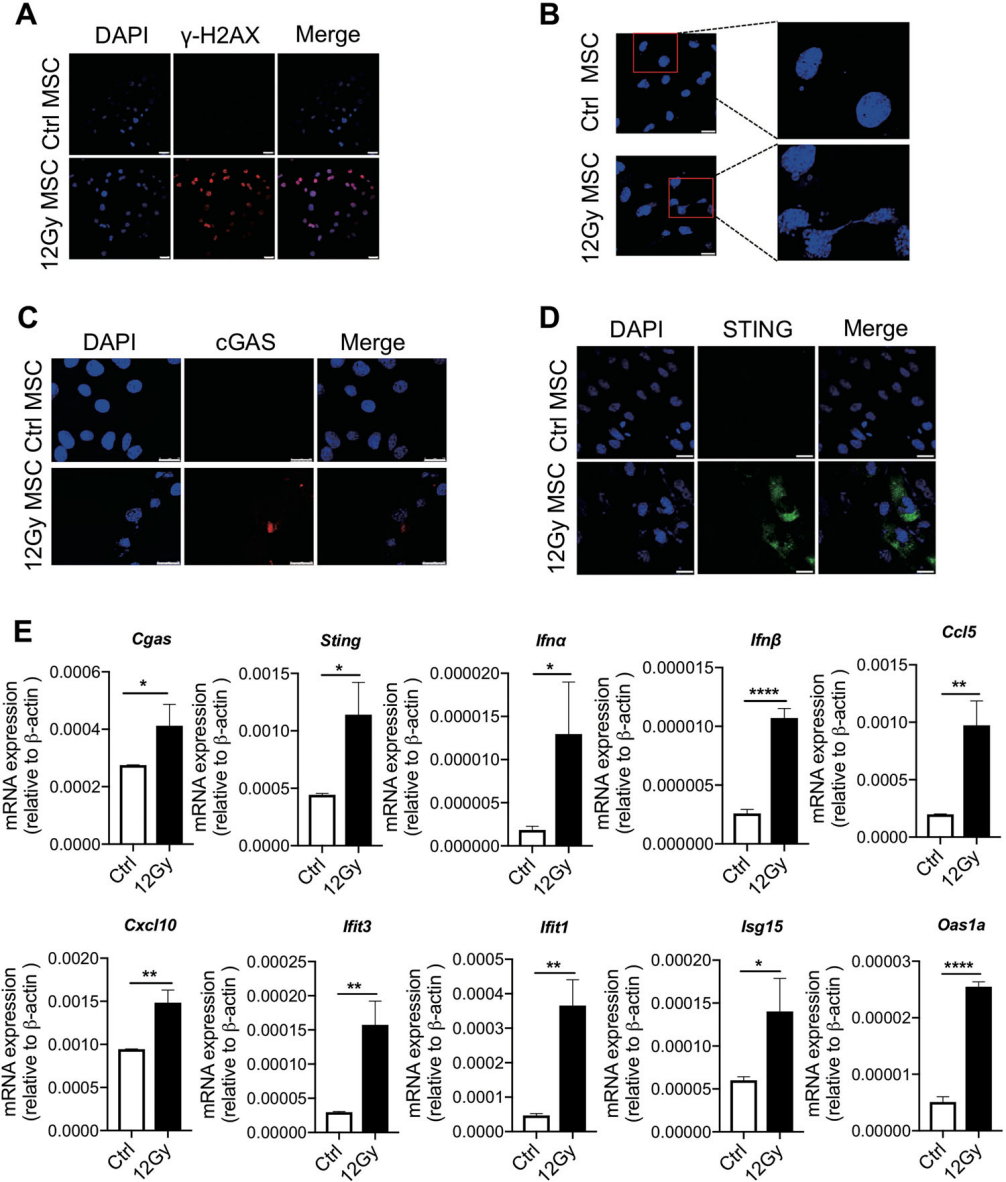
cGAS–STING pathway is activated in irradiated MSCs. **a** MSCs were subjected to immunofluorescence staining with γ-H2AX antibody at 2 h after irradiation (12 Gy); scale bar, 25 µm. **b** MSCs were irradiated (12 Gy) for 48 h, staining with DAPI. Nuclei and micronuclei are shown; scale bar, 25 µm. **c**, **d** The expression levels of cGAS(C) and STING(D) were detected by immunofluorescence; scale bar (**c**), 75 µm; scale bar (**d**), 75 µm. **e** MSCs were irradiated (12 Gy) and cultured for 24 h, and then the expression level of ISGs were determined by real-time PCR. All experiments in this figure were repeated at least three times. *p < 0.05, **p < 0.01, and ****p < 0.0001.

IR may induce many types of DNA damage including double-strand breaks (DSBs). We observed that the level of γ-H2AX, a marker for DSBs, was significantly increased in irradiated MSCs (Fig. 3a). In addition, micronuclei, which may contain immunostimulatory DNA, were abundantly induced by IR (Fig. 3b). It was recently reported that cGAS can sense and is activated by cytosolic double-stranded DNA (dsDNA)^6,12^. cGAS activation generates the cyclic dinucleotide cyclic GMP–AMP (cGAMP), which in turn induces a type I interferon response via the adaptor STING^8^. Indeed, immunofluorescence staining confirmed the activation of cGAS and STING by IR (Fig. 3c, d). Consistently, interferon-stimulated genes (ISGs), the major downstream component of cGAS–STING signaling, were upregulated upon radiation (Fig. 3e), suggesting that innate immune signaling in MSCs is activated by IR.

### Blockage of cGAS–STING signaling abolishes the pro-metastatic effect of irradiated MSCs

**Fig. 4:**
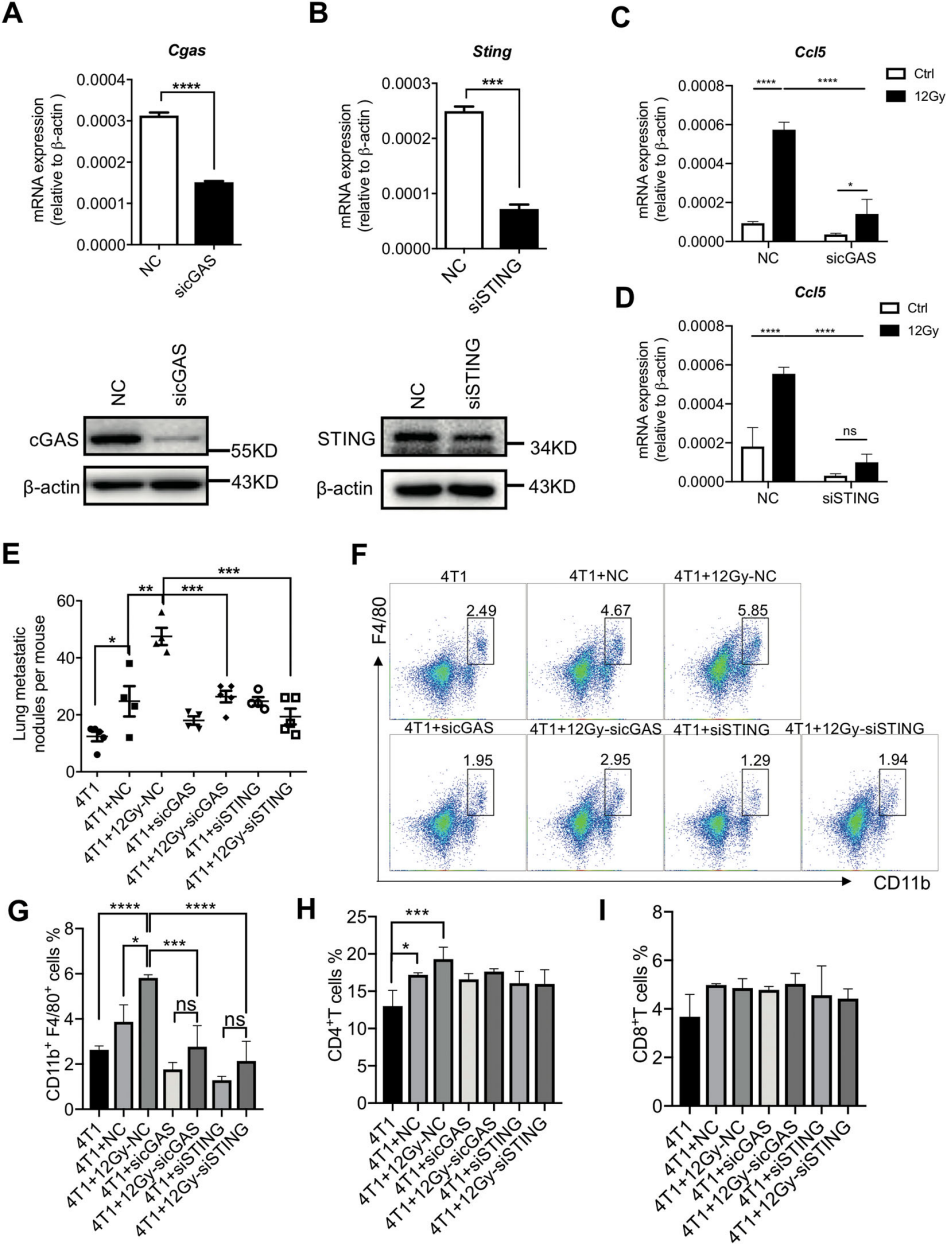
cGAS–STING signaling is required for the pro-metastatic effect of irradiated MSCs. **a** MSCs were transfected with siRNA for 24 h and then cultured for another 24 h after irradiation (12 Gy). The expression levels of cGAS in MSCs were determined by real-time PCR. MSCs were transfected with siRNA for 24 h and then cultured for another 48 h after irradiation (12 Gy). The expression levels of cGAS were analyzed by western blotting. **b** MSCs were transfected with siRNA for 24 h and then cultured for another 24 h after irradiation (12 Gy). The expression levels of STING in MSCs were determined by real-time PCR. MSCs were transfected with siRNA for 24 h and then cultured for another 48 h after irradiation (12 Gy). The expression levels of STING were analyzed by western blotting. **c**, **d** MSCs transfected with sicGAS or siSTING for 24 h were cultured for another 24 h after irradiation (12 Gy). The expression level of Ccl5 was determined by qPCR. **e**, **i** MSCs (1 × 10^4^) were transfected with siRNA for 24 h and then cultured for another 24 h after irradiation (12 Gy). These cells were co-injected with 4T1 cells (5 × 10^4^) into BALB/c mice via tail vein. Metastatic nodules were counted after 14 days (**e**). Single-cell suspensions prepared from lung tissues were analyzed for the frequency of CD11b^+^F4/80^+^ macrophages (**f**–**g**), CD4^+^ T cells (**h**) and CD8^+^ T cells (**i**) by flow cytometry. *n* ≥ 3 for each group. All experiments in this figure were repeated at least three times. *p < 0.05, **p < 0.01, ***p < 0.001, and ****p < 0.0001; ns not significant.

To investigate whether cGAS–STING signaling mediated the metastasis-promoting effect of irradiated MSCs, we depleted cGAS and STING respectively using siRNAs (Fig. 4a, b). As expected, either cGAS or STING knockdown remarkably attenuated irradiation-induced expression of ISGs, indicating that the cGAS–STING axis had a key role in IR-induced activation of innate immune signaling (Fig. 4c, d, Supplementary Figs. S1 and S2). Importantly, when cGAS or STING was depleted, the pro-metastatic effect of MSCs endowed by IR was abolished (Fig. 4e). These results demonstrate that the activation of cGAS–STING signaling mediated the metastasis-promoting effect of irradiated MSCs.

We next studied how the cGAS–STING signaling pathway mediates the metastasis-promoting effect of MSCs. As shown in Fig. 3e, IR could transactivate a variety of type I interferon-related genes, including those encoding chemokines Ccl5 and Cxcl10. However, of the two chemokines, only the upregulation of Ccl5, but not that of Cxcl10, induced by IR was impaired by cGAS or STING depletion (Fig. 4c, d, Supplementary Figs. S1 and S2). Considering that CCL5–CCR5 axis is critical for the recruitment of T cells and macrophages, we examined the immunocytes by flow cytometry and found that irradiated control MSCs, but not irradiated sicGAS or siSTING MSCs, could significantly increase the accumulation of macrophages in the lung (Fig. 4f, g). However, they had no such an effect on the accumulation of CD4^+^ T cells or CD8^+^ T cells (Fig. 4h, i). These data indicate that the activation of cGAS–STING signaling mediated the recruitment of macrophage to the lung.

### Macrophages are essential for the metastasis-promoting effect of irradiated MSCs

**Fig. 5:**
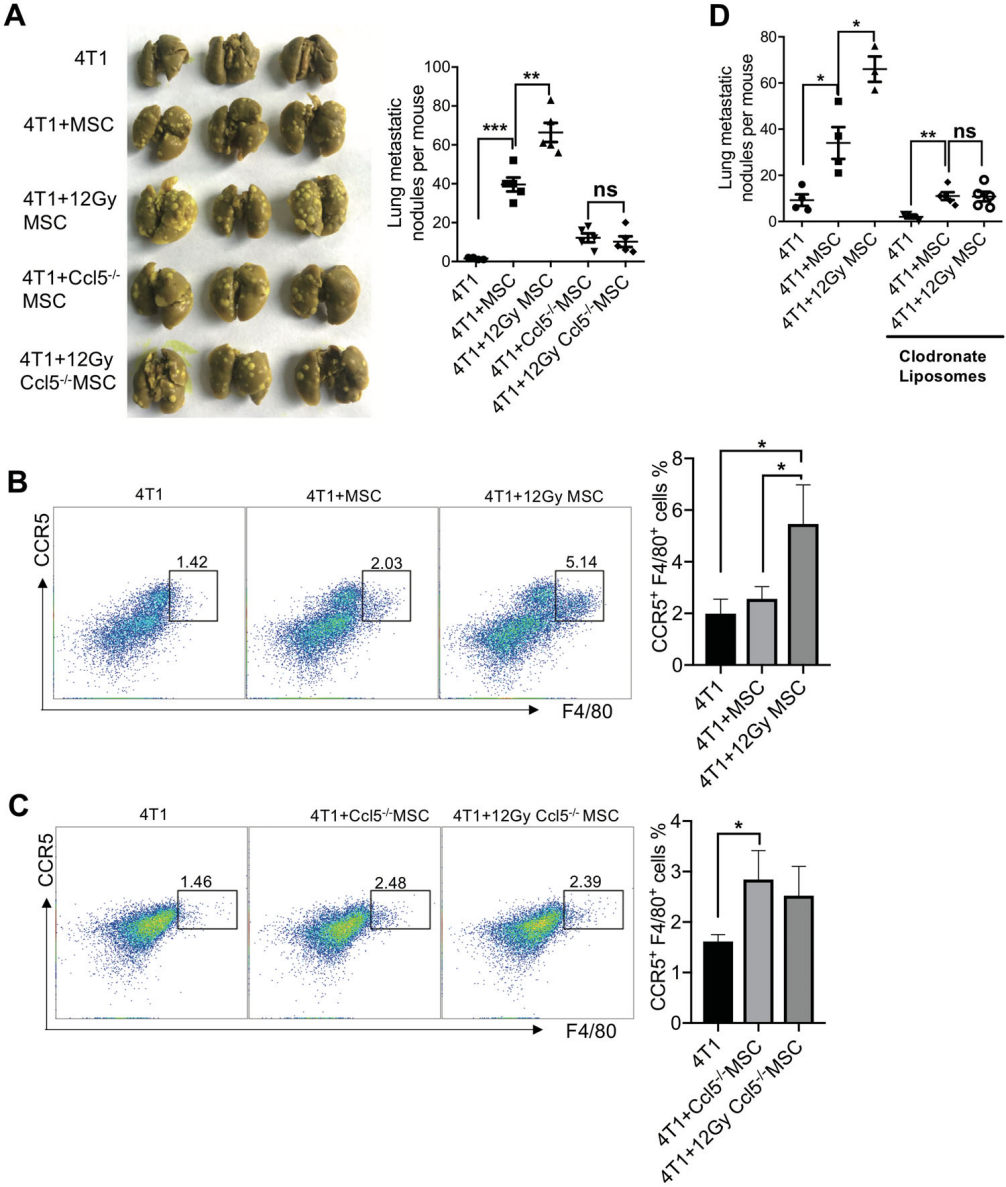
Upregulation of CCL5 in irradiated MSCs mediates increased lung metastasis. **a** MSCs (1 × 10^4^) or *Ccl5*-deficient MSCs (1 × 10^4^) were irradiated (12 Gy) and cultured for 24 h before co-injected with 4T1 cells (5 × 10^4^) into BALB/c mice via tail vein. Metastatic nodules were counted after 14 days. *n* ≥ 4 for each group. **b** MSCs (1 × 10^4^) or irradiated (12 Gy) MSCs (1 × 10^4^) were co-injected with 4T1 cells (5 × 10^4^) into BALB/c mice via tail vein. Single-cell suspensions prepared from lung tissues were analyzed for the frequency of CCR5^+^F4/80^+^ macrophages by flow cytometry after 14 days. *n* = 3 for each group. **c** *Ccl5*-deficient MSCs (1 × 10^4^) or irradiated (12 Gy) *Ccl5*-deficient MSCs (1 × 10^4^) were co-injected with 4T1 cells (5 × 10^4^) into BALB/c mice via tail vein. Single-cell suspensions prepared from lung tissues were analyzed for the frequency of CCR5^+^F4/80^+^ macrophages by flow cytometry after 14 days. *n* = 4 for each group. **d** MSCs (1 × 10^4^) or irradiated (12 Gy) MSCs (1 × 10^4^) were co-injected with 4T1 cells (5 × 10^4^) into BALB/c mice via tail vein. Clodronate liposomes were injected (2 mg/kg, i.p.) to mice every three days. Metastasis lung tumor nodules were counted after 14 days. *n* ≥ 3 for each group. All experiments in this figure were repeated at least three times. *p < 0.05, **p < 0.01, ***p < 0.001; ns not significant.

Next, we investigated whether Ccl5 mediated the pro-metastatic effect of irradiated MSCs by using MSCs derived from Ccl5 null mice. *Ccl5*^−/−^ MSCs were less effective in promoting lung colonization than wild-type MSCs. Importantly, the *Ccl5*^−/−^ MSCs no longer exhibited an increased metastasis-promoting ability as the wild-type MSCs did when irradiated (Fig. 5a). These results suggested that the pro-metastatic property of MSCs upon irradiation depended on CCL5. Furthermore, we examined the immunocytes and found that when infused via tail vein, irradiated wild-type MSCs could significantly increase the accumulation of CCR5^+^ macrophages in the lung (Fig. 5b). In contrast, no such increase was detected when irradiated *Ccl5*-deficient MSCs were similarly transferred (Fig. 5c). These data indicate that CCL5–CCR5 axis mediates the enhanced infiltration of macrophages caused by irradiated MSCs. Moreover, when macrophages were depleted with CL-liposomes, the irradiated MSCs were no longer capable of promoting metastasis (Fig. 5d).

**Fig. 6:**
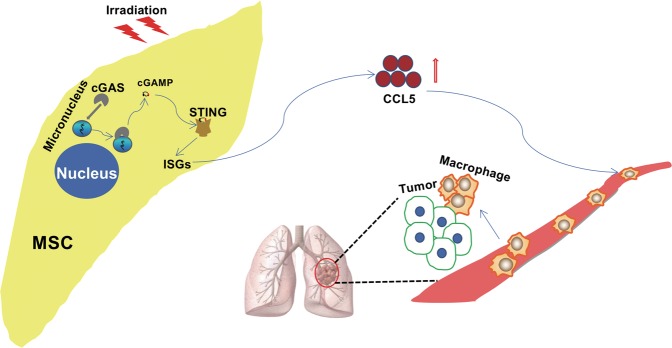
A schematic model for the pro-metastatic effect of irradiated MSCs. X-ray induces DNA damage in MSCs and activates cGAS–STING pathway, and consequently the downstream ISGs, including chemokine CCL5. Upregulated CCL5 in MSCs promotes the recruitment of macrophages into lung, and facilitates the colonization of tumor cells.

Altogether, these data demonstrate that cGAS–STING signaling in MSCs are activated by IR and drives the production of CCL5, which can remodel the lung microenvironment via recruiting macrophages that are essential for the colonization of cancer cells in the lung (Fig. 6).

## Discussion

In this study, we demonstrated that irradiated MSCs acquire an enhanced capacity in promoting breast cancer metastasis to non-irradiated lungs. The irradiated MSCs highly express CCL5 upon the activation of cGAS–STING signaling. The upregulated CCL5 production is responsible for the increased recruitment of macrophages into the lung, which subsequently facilitates the lung colonization of breast cancer cells. These data demonstrate a novel crosstalk between MSCs and immune cells that is initiated by irradiated MSCs via the activation cGAS–STING signaling.

It is known that inflammation is activated in the metastatic niche, which comprises many types of activated immune cells^13^. Even before metastasis occurs, secondary sites favorable for the seeding and colonization of disseminated cancer cells, also known as pre-metastatic niches, are already established by the primary tumors^14–16^. Importantly, inflammatory cytokines or chemokines were found to be critical for recruiting bone marrow-derived cells and tumor cells to pre-metastatic sites^15^. Extracellular matrix protein versican derived from Lewis lung carcinoma was found to activate bone marrow-derived macrophages by binding to TLR2, leading to secretion of inflammatory cytokines such as TNF-α to promote metastasis^16^. Our work demonstrated that cGAS–STING signaling was activated in irradiated MSCs, which led to the upregulation of CCL5 and consequently the increased recruitment of pro-metastatic macrophages into the lung. Together, these findings demonstrate a critical role of the inflammatory cells and factors in the pre-metastatic niches.

Macrophages are highly plastic and are recognized as key mediators of tumor progression. Our present findings demonstrate that irradiated MSCs could recruit macrophages via CCL5 and promote tumor metastasis to non-irradiated organs. Furthermore, CD11b^+^F4/80^+^ macrophages were found to be pivotal for the metastasis-promoting activity of irradiated MSCs. Blockage of such interactions might be a useful strategy for treating stromal cell-related diseases.

Innate immune response induced by DNA damage via cGAS–STING axis results in the expression of type I interferons and the transcriptional activation of more interferon-stimulated genes (ISGs). However, opposite roles of cGAS–STING signaling in cancer development and metastasis have been reported. On one hand, cGAS–STING-mediated IFN production can be amplified by infiltrating lymphocytes to promote antitumor immunity^17^. STING-deficient mice were shown to be compromised in their response to IFN γ due to the reduction of tumor-specific CD8^+^ T cells^17^. cGAS–STING pathway is also involved in radiation-mediated antitumor immunity via dendritic cell sensing of irradiated-tumor cells^18^. On the other hand, cGAS–STING axis has also been implicated in tumor metastasis. Chromosomal instability was shown to drive metastasis of breast cancer via activation of cGAS–STING signaling^9^. Transfer of cGAMP from cancer cells to astrocytes via carcinoma–astrocyte gap junctions was shown to activate the STING signaling in astrocytes, which consequently produce paracrine signals to activate the pro-survival pathways in brain metastatic cells^19^. Our study indicated that MSCs displayed a stronger metastasis-promoting property when treated with irradiation. Thus, blockage of cGAS–STING axis both in stromal cells and tumor cells could be a potential anti-metastasis therapy. In addition, neutralization of CCL5 may help to reduce the risk of lung metastasis in cancer patients under radiotherapy.

Accidental local exposure to IR or localized cancer radiotherapy may promote cancer metastasis to non-irradiated organs. Several clinical studies have reported that radiation therapy could contribute to the distant metastases via increasing the number of circulating tumor cells in bladder cancer^20^ and lung cancer^21,22^. Notably, radiation therapy resulted in a high risk of metastases to non-irradiated organs in these tumor types^23^. Our data showed that localized radiation of tumor mass led to increased metastasis to un-irradiated lung, indicating that the local radiation may have altered the pulmonary microenvironments. Furthermore, irradiated MSCs injected via tail vein could promote the pulmonary colonization of cancer cells. Therefore, MSCs could act as a mediator of IR-induced metastasis.

MSCs-based therapies are being actively tested for the treatment of many different diseases^24,25^. Our findings presented here suggest that cautions should be taken when preparing MSCs for clinical use, MSCs that are aged or are otherwise compromised in genomic integrity have the potential to further increase metastasis of hidden tumors. Considering that there is a lack of general criteria for selecting MSCs for clinical applications^26,27^, greater attention to the qualities of MSCs should be paid.

In conclusion, our data indicate that locally irradiated MSCs may contribute to cancer metastasis to distant organs. Circulating MSCs with activated cGAS–STING–CCL5 axis can reconstitute a tissue microenvironment that is more conducive to disseminated cancer cells.

## Materials and methods

### Animals

Female BALB/c mice were purchased from Suzhou Laboratory Animal Center, Soochow University, Jiangsu, China. All animals were maintained under pathogen-free conditions in the Vivarium of Soochow University. All animals were 6–8 weeks old in each experiment. The animal experimental procedures in this study were approved by the Laboratory Animal Ethics Committee of Soochow University. No specific statistical method was applied to determine the mouse number.

### Cell line

Murine 4T1, mammary tumor cells, from mammary gland of BALB/cfC3H mice, were cultured in Dulbecco’s modification of Eagle’s medium (DMEM) high supplemented with 10% bovine serum albumin (FBS), 2 mM glutamine, 100 U/ml penicillin and 100 g/ml streptomycin (all from Invitrogen, USA).

### Cell cultures

Mouse MSCs were cultured in Dulbecco’s modification of Eagle’s medium (DMEM) low with 10% fetal bovine serum (FBS), 2 mM glutamine, 100 U/ml penicillin and 100 μg/ml streptomycin (all from Invitrogen, USA) at 37 °C in a humidified incubator supplemented with 5% CO_2_. 4T1 cells were cultured in DMEM High with 10% fetal bovine serum, 2 mM glutamine, 100 U/ml penicillin and 100 μg/ml streptomycin.

### RNA oligoribonucleotides

Small interfering RNA (siRNA) duplexes were purchased from GenePharma.

siSTING-1 Sense strand: 5′-GAUUCUACUAUCGUCUUAUTT-3′, Antisense strand: 5′-AUAAGACGAUAGUAGAAUCTT-3′;

siSTING-2 Sense strand: 5′-GCAUCAAGAAUCGGGUUUATT-3′, Antisense strand: 5′-UAAACCCGAUUCUUGAUGCTT-3′;

sicGAS-1 Sense strand: 5′-GGCCGAGACGGUGAAUAAATT-3′, Antisense strand: 5′-UUUAUUCACCGUCUCGGCCTT-3′;

sicGAS-2 Sense strand: 5′-GGAAAUCCGCUGAGUCAUUTT-3′, Antisense strand: 5′-AAUGACUCAGCGGAUUUCCTT-3′.

### Cell transfections

siSTING is composed of siSTING-1 and siSTING-2. sicGAS is composed of sicGAS-1 and sicGAS-2. Reverse transfection of RNA oligoribonucleotides were performed using Lipofectamine RNAiMAX (Life Technologies).

### Irradiation procedure with X-rays

#### Cells

MSCs were irradiated with 12 Gy (at a dose rate of 1.5 Gy/min) X-rays. X-rays were administered by Animal X-ray Irradiator (X-RAD 320ix, USA) in Soochow University. Control cells were removed from the incubator at the same time without radiation exposure. Cells were returned to the incubator for 24 or 48 h after irradiation.

#### Animals

Mice were irradiated with 4 Gy (at a dose rate of 1.5 Gy/min) X-rays. X-rays were administered by Animal X-ray Irradiator (X-RAD 320ix, USA) in Soochow University. Control group were removed from the mouse colony at the same time without radiation exposure.

### Reagents

Bouin’s solution was from Sigma-Aldrich (HT10132-1L). Negative control (NC) and small interfering RNA (siRNA) were purchased from GenePharma (Shanghai, China). Mouse cGAS (31659S) and GAPDH (5174S) monoclonal Abs were obtained from Cell Signaling Technology (Beverly, MA, USA). STING (ab181125) monoclonal Abs were obtained from Abcam. SYBR Green reagent was obtained from ThermoFisher Scientific (New York, USA). CD11b (11-0112-82), Ly6G (12-5931-83), Sca1 (12-5981-83), CD29 (12-0291-82), CD44 (12-0441-83), CD34 (13-0341-82), CD45 (12-0451-82) and CD31 (12-0311-82) antibody and isotype control antibody (12-4714-81) were purchased from ThermoFisher Scientific (New York, USA). F4/80 (123114), CCR5 (107016) and CD8 (100712) were purchased from Biolegend (San Diego, CA, USA). CD4 (552051) was purchased from BD Biosciences.

### Animal studies

#### Lung colonization model

Mice were randomly allocated to different groups. 4T1 cells (5 × 10^4^) were co-injected with MSCs or irradiated MSCs (1 × 10^4^) into BALB/c mice via tail vein injection. After 2 weeks, the immune cells in lungs were analyzed by flow cytometry (Cytoflex, Beckman Coulter), and tumor nodes on the lung were counted via Bouin’s solution staining using double blind method.

#### Metastasis model

Mice were randomly allocated to different groups. BALB/c mice were subcutaneously injected with 4T1 cells (4 × 10^5^), 10 days later the tumor sites were irradiated (4 Gy) with X-ray. The tumor volume and metastatic nodules were recorded after 30 days using double blind method.

### Quantitative real-time PCR

Total RNA was isolated using RNAprep pure Cell Kit (Feijie Biotech, Shanghai, China). cDNA was reverse-transcribed using the PrimeScript™ RT Master Mix (TaKaRa Biotech, RR036A). The mRNA levels were quantified by real-time PCR (ABI Quant Studio 6, Life) with SYBR Green Master Mix (ThermoFisher Scientific, USA). The total amount of mRNA was compared with endogenous β-actin mRNA. Sequences of PCR primer pairs were listed in Supplementary Table S1.

### Western blotting

MSCs were washed with ice-cold phosphate buffered saline (PBS) and lysed in RIPA buffer. The concentration of total protein was determined with BCA Protein Assay Kit (ThermoFisher scientific, 23227). Protein samples were separated using 10% SDS-PAGE and transferred to PVDF membranes. The membranes were blocked in TBST containing Tween 20 with 5% bovine serum albumin (BSA) at room temperature for 1 h, then the primary antibodies were incubated overnight at 4 °C. The membrane was washed three times in TBST (5 min each), then incubated with secondary antibody at room temperature for 1 h. The membrane was washed three times and then proteins were detected by enhanced chemiluminescence (Beyotime, Shanghai, China).

### Immunofluorescence

MSCs were seeded in a 12-well plate and accepted irradiation with 12 Gy X-rays. After 48 h, cells were fixed in 4% PFA in PBS for 20 min at room temperature and washed with PBS. Cells were incubated in PBS containing 3% BSA and 0.1% Triton X-100 for 2 h at room temperature. Cells were then incubated with primary antibody overnight at 4 °C and then with a secondary antibody for 1 h. Cell nucleus was subsequently stained with DAPI, and was imaged under a Laser scanning Confocal Microscopy (Leica Biosystems).

### Depletion of macrophages

4T1 cells (5 × 10^4^) were co-injected with MSCs or irradiated MSCs (1 × 10^4^) into BALB/c mice via tail vein injection. Clodronate Liposomes/PBS were injected (2 mg/kg, i.p.) to mice every 3 days. Metastasis lung tumor nodules were counted after 14 days.

### Flow cytometric analysis

Cells were quantified by flow cytometry using anti-CD11b, anti-F4/80, anti-CCR5, anti-Ly6G, anti-CD4 and anti-CD8. Cells were harvested after type II collagenase (ThermoFisher 17101015) in DMEM for 60 min at 37 °C, went through a 70 μM cell strainer, and resuspended in PBS supplemented with 2% FBS. Then cells from each well were suspended in 50 μl staining buffer (PBS containing 2% FBS) containing the indicted monoclonal antibodies and incubated for 20 min at 4 °C. Finally, cells were washed twice and resuspended in 200 μl of PBS, and then analyzed on a flow cytometer (Cytoflex, Beckman Coulter).

### Statistical analysis

All data were obtained from at least three independent biological replications and were reported as means ± SEM. ns, not significant; *p < 0.05, **p < 0.01, ***p < 0.001 and ****p < 0.0001 by unpaired and two-tailed Student’s *t*-test when only two groups were compared or ANOVA test when more than two groups were compared using the GraphPad Prism software (GraphPad Software, Inc., San Diego, CA, USA). All analyses were carried out on normally distributed data.

## Supplementary information

supplementary figure legends

supplementary figure 1

supplementary figure 2

supplementary table 1
