# Supplementary material for: Irradiation induces cancer lung metastasis through activation of the cGAS–STING–CCL5 pathway in mesenchymal stromal cells
Source: Cell Death Dis. 2020 May 7;11(5):326. doi: 10.1038/s41419-020-2546-5 (PMC7206094; doi:10.1038/s41419-020-2546-5)
Supplement: Supplementary file 1 — supplementary figure legends [file 41419_2020_2546_MOESM1_ESM.docx]

**Supplemental Figure legends**

**Figure S1. cGAS is involved in IR-induced activation of innate immune signaling.**

MSCs transfected with sicGAS for 24 h were cultured for another 24 h after irradiation (12 Gy). The expression level of ISGs were determined by qPCR. Data are expressed as means ± SEM. All experiments in this Figure were repeated at least three times. *p < 0.05, **p < 0.01, ***p < 0.001; ns, not significant.

**Figure S2. STING is involved in IR-induced activation of innate immune signaling.**

MSCs transfected with siSTING for 24 h were cultured for another 24 h after irradiation (12 Gy). The expression level of ISGs were determined by qPCR. Data are expressed as means ± SEM. All experiments in this Figure were repeated at least three times. *p < 0.05, **p < 0.01, ***p < 0.001; ns, not significant.
