# Supplementary figures and images for: Irradiation induces cancer lung metastasis through activation of the cGAS–STING–CCL5 pathway in mesenchymal stromal cells
Source: Cell Death Dis. 2020 May 7;11(5):326. doi: 10.1038/s41419-020-2546-5 (PMC7206094; doi:10.1038/s41419-020-2546-5)

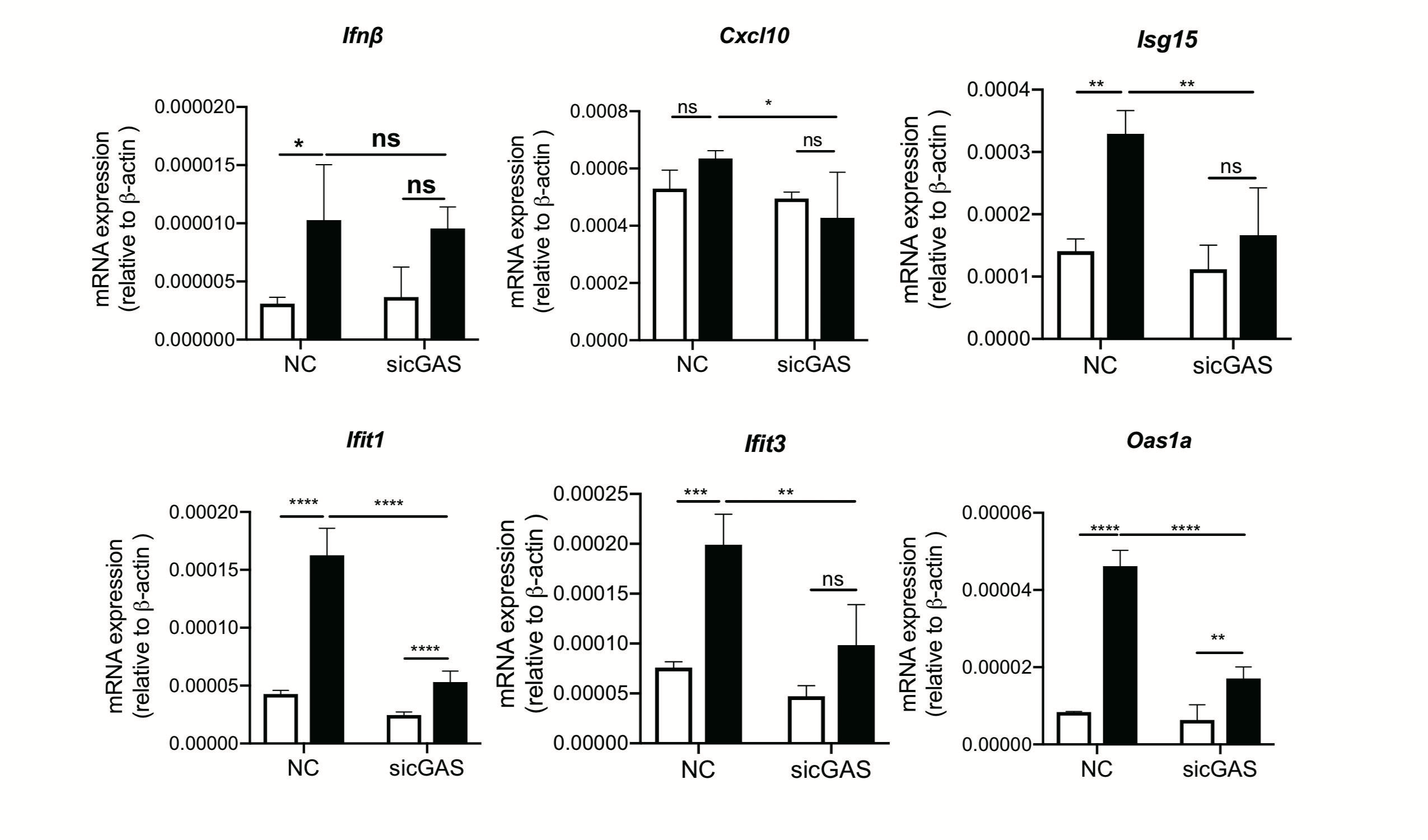

Supplement: Supplementary file 2 — supplementary figure 1 [file 41419_2020_2546_MOESM2_ESM.png]

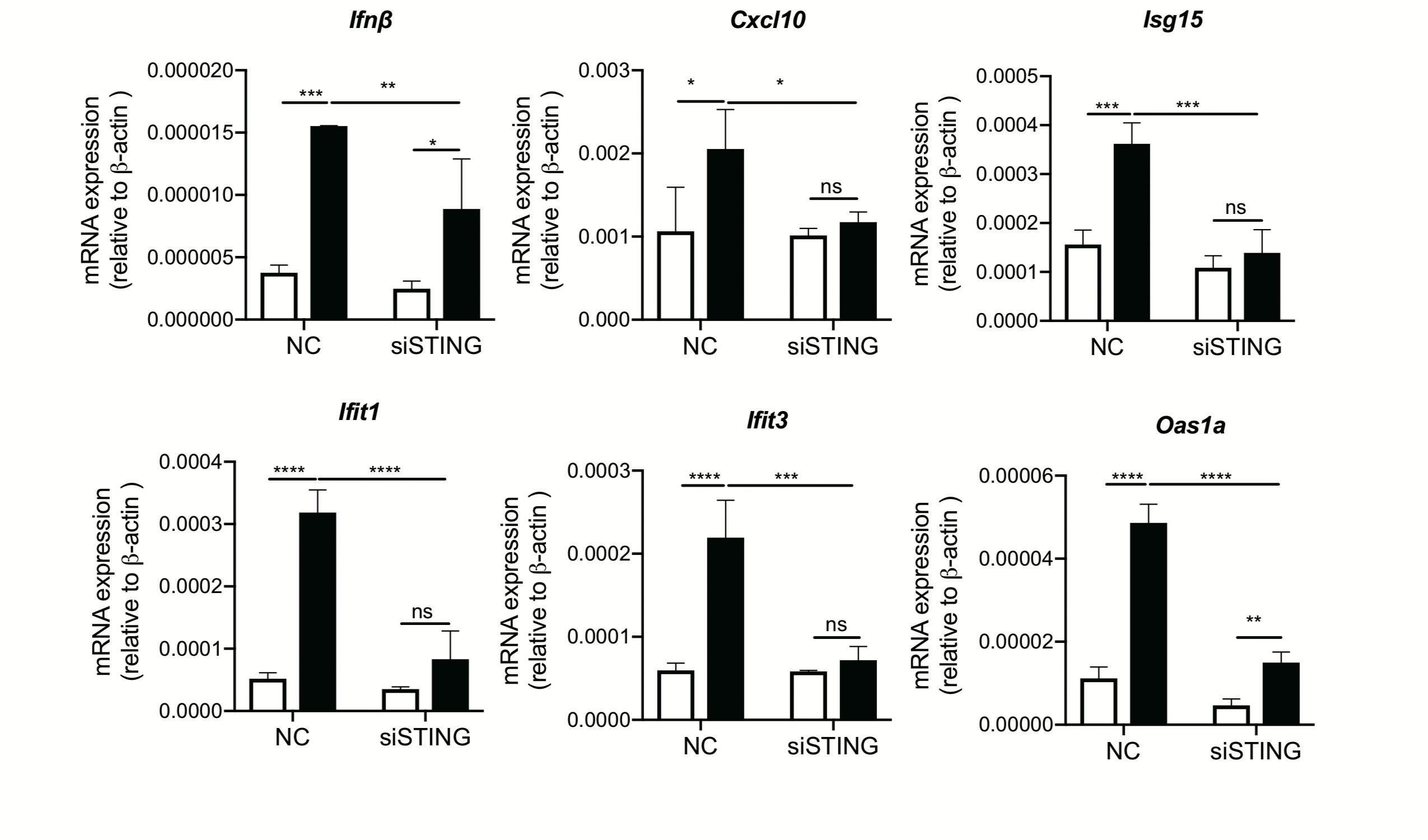

Supplement: Supplementary file 3 — supplementary figure 2 [file 41419_2020_2546_MOESM3_ESM.png]
